# Evaluating the Use of Balint Groups in Medical Student Psychiatric Education

**DOI:** 10.1192/bjo.2025.10273

**Published:** 2025-06-20

**Authors:** Adama Fullah, Harvey Markham, James Fallon, Poppy Riddle

**Affiliations:** 1 Brighton and Sussex Medical School, Brighton, United Kingdom; 2 University of Sussex, Brighton, United Kingdom; 3 Sussex Partnership NHS Foundation Trust, Worthing, United Kingdom

## Abstract

**Aims:** This qualitative research project examined the attitudes of third-year medical students to a new, weekly, one-hour, mandatory online Balint group during 4 weeks of their Psychiatry rotation.

**Methods:** All BSMS Year 3 students participated in 4 Balint group sessions as a compulsory part of their 5-week psychiatry rotation within a 10-week module. 193 students in the 2021–22 academic year took part in Balint groups as part of their formal psychiatry teaching. 81 participants completed part or all of the post-intervention questionnaire, which included free-text and Likert scale ratings. Thematic analysis of post-intervention free-text responses was conducted by three independent researchers.

**Results:** Four themes were identified.

Firstly, “Balint groups as a positive experience” with 86% (n=55 of 64) of respondents reporting they would consider attending Balint groups again as a medical student and 86% (n=53 of 61) that they would attend as qualified doctors. Students generally reported that they found Balint groups useful as a means to reflect upon clinical encounters.

Theme 2 was “Balint groups as a way to change clinical practice”, students described developing a greater understanding of how emotions may impact upon the clinical encounter. Within this theme, the subtheme of “Coping in clinical practice” emerged, with students reporting that Balint groups helped them manage feelings of isolation and improved reflective skills.

Theme 3 was “Balint groups as a way to explore perspectives”. Respondents reflected that Balint groups allowed them to explore different dimensions of the doctor-patient relationship. This included accepting that doctors may be impacted emotionally by patients and that the emotions of both the patient and the doctor can affect or challenge the clinical encounter and relationship.

Theme 4 centred around “Barriers to the experience”, with recurrent themes of time pressure, fear of being judged by others and some feelings that Balint groups were not relevant to their practice. Within this theme, some students seemed to misunderstand the aims of Balint groups. For example, some students wished that concrete techniques and 'coping strategies’ had been taught, a subtheme of an “expectation/reality mismatch”.

**Conclusion:** Our results show that students found the Balint group both well tolerated and useful. However, notably few mentioned the doctor-patient relationship in their feedback, despite it being the core aim of the Balint group. Our research shows that while Balint groups can benefit students in various ways, further work may be needed to help students understand their scope and purpose.

